# Lichen Simplex Chronicus That Accompanies Anogenital Warts during the Childhood

**DOI:** 10.1155/2012/192767

**Published:** 2012-10-17

**Authors:** Özlem Seçilmiş Kerimoğlu, Nasuh Utku Doğan, Aybike Tazegül, Mehtap Karameşe, Hasan Beyhekim, Çetin Çelik

**Affiliations:** Obstetrics and Gynecology Department, Selçuklu Medicine Faculty, Selçuk Univercity, Konya, Turkey

## Abstract

Anogenital warts and lichen simplex chronicus (LSC) are rarely seen during the childhood. A 9-year-old girl has been presented to hospital by her parents with itching in the anogenital area. There were anogenital warts and a different erythematous lesion in the perianal region. On the pulpa of the right thumb, there was a wart extending under the nail. The lesions are surgically removed. The results of the histopathological examination were reported as condyloma acuminata and LSC. Children with anogenital warts should be examined carefully to discover the transmission route and other possible concomitant cutaneous diseases.

## 1. Introduction

Anogenital lesions observed during the childhood may result from infections, trauma, skin diseases, or neoplasia, as well as injuries and infections caused by sexual abuse. As among anogenital lesions, anogenital warts resulting from human papilloma virus (HPV) infection are one of the most commonly seen sexually transmitting diseases among the adults. Sexual abuse should be considered when it is detected during the childhood. Clinicians experience a big difficulty for the determination of its transmission route in this age group. Although HPV infection may be transmitted via sexual abuse or sexual route, it should be kept in mind that it may show a nonsexual transmission route as well, especially in the pediatric population [1]. 

Lichen simplex chronicus (LSC) is a chronic inflammatory disease which shows severe itching. While LSC is a disease generally seen in the adulthood, it is a cutaneous lesion rarely seen during the childhood, mostly observed in the male children [2]. In the cases of primary LSC, it is known that emotional status is very efficient in the predisposed people, and that it may develop as a secondary manifestation in the cutaneous diseases that lead to itching or in HPV infections, candidiasis, or tinea cruris [2, 3].

In this case report, we aimed to focus on a girl diagnosed with anogenital warts related to HPV, which is a rarely seen infection in the childhood, and with LSK, a rarely seen cutaneous disease in that age group, to evaluate the concomitant presence of these two conditions and to highlight the potential nonsexual transmission routes of HPV.

## 2. Case Report

A 9-year-old girl has been presented to our hospital by her parents with anogenital pruritus and with lesions detected in this area by her mother. It was learned that she was the only child of the family, and her personal care was provided by her mother. Her personal history and familial history were normal.

Her mother told that pruritus had begun approximately one month ago, and at that time, there has been only one single lesion in the perianal area, but the number of these lesions had increased over time, and she had experienced itching even during the sleep for the last 3 months. We learned that the child has been taken to another center for a visit with the same findings 15 days before, and there, she has received cryotherapy, but there has been no regression in the complaints and in the lesion size.

 In the examination, there were skin-colored warts with the sizes of 3 × 3 mm, 2 × 4 mm ve 10 × 14 mm in three different areas of the perianal region, and of 13 × 12 mm in the right groin. In the right side of the perianal region, there was another area of 10 × 16 mm, slightly more protuberant compared to skin level, with marked margins and erythema, which was different from other lesions. on whole body examination, no sign of trauma was seen and inspection of the anogenital region did not reveal a sign of sexual abuse. On the pulpa of the right thumb, there was a white-gray, hyperkeratotic wart that extended under the nail. It was learned that this wart has been formed approximately 1.5 years before, and the patient received cryotherapy for this wart and anogenital lesions in an external center.

For the patient who has been evaluated for condylomata acuminata and warts by the department of dermatology and for sexual abuse by the department of forensic medicine, it was stated that the warts detected in the anogenital region could be developed secondarily to the autoinoculation from the contact of hand genitalia. In the interviews and examination, no sign of sexual abuse was obtained. Mother and father had no history of wart.

As the patient has undergone cryotherapy in another center, we waited for one month to evaluate the response given to this previous therapy, but we decided to give a therapy due to few regression of the lesions and excessive increase of itching. Due to the failure of previous destructive therapy, surgical excision was considered to be appropriate, and the lesions were excised under anesthesia and sampled for the histopathological examination. We also take biopsy from the protuberant lesion with marked margins and erythema in the right of the perianal region. The results of the histopathological examination of the surgically removed warts were reported as condyloma acuminate, and the histopathological examination revealed LSK for erythematous area biopsy. No additional therapy was considered to be required for the wart detected on the thumb. For the treatment of LSK, topical steroids, which are administered as a standard therapy, were not preferred for this patient due to the underlying disease described as HPV infection-related wart. For the palliation of the episodes of itching, we begun to give hydroxyzine hydrochloride, a sedative antihistaminic, at a dose of 10 mg/day via oral route, and the parents and the child were instructed to protect the hygiene of the anogenital area and to avoid the contact of hand-genitalia.

During the 2-month followup, no additional lesion was observed in the anogenital area, it was seen that itching episodes were gradually decreased and disappeared, and as a result of the histopathological examination, it was revealed that the area with LSK-like erythema in the right side of the perianal area disappeared. It was determined that the size of the wart localized on the right thumb was reduced, but it did not disappear, and that no additional therapy was planned for the treatment of the lesion.

**Figure 1:**
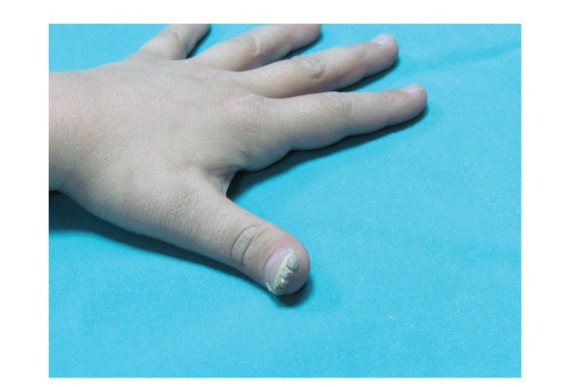
Wart under the right thumb nail.

The lesions located in the anogenital region and on the thumb were photographed to include in the forensic file, but the parents consented only the use of the photograph of the thumb lesion in this case report (Figure 1). 

## 3. Discussion

While sexual abuse is a transmission route that should be evaluated when investigating the transmission route of HPV in anogenital warts detected during the childhood, it is known that it may be vertically transmitted from infected mother during the pregnancy or during the delivery, via direct contact with genital HPV infection or other warts present in the parents or via autoinoculation from the warts located on the hand or in the other parts of the body of the child [4]. 

While the most common causal factors for anogenital warts in adults and in adolescents were mucosatropic HPVs types 6 and 11, it was reported to be both mucosatropic HPVs types 6 and 11 and cutaneotropic HPVs types 1 and 2 in children [5–7]. Although HPV typing provides an insight about the transmission route during the clarification of the transmission route, etiology cannot be definitively revealed. Even if anogenital warts are caused by HPV types 1 or 2, it may indicate an autoinoculation or it might be caused by an infection developed as a result of direct contact without a sexual abuse or of a contact or digital penetration related to sexual abuse. Therefore, viral typing is not recommended as a routine method in this condition [4, 8]. 

Whole body examination should be done by seeking for the signs of sexual abuse; anogenital warts should be individually described, and the presence of oral warts should be investigated and, if possible, photographed. Parents should be interrogated for the presence of anogenital warts or of warts in other parts of the body, and if a positive history exists, examination should be recommended [4]. Especially in the children aged above 3 years old, when anogenital warts are detected, forensic medicine specialists trained about sexual abuse should make an interview with the patient, and, during this interview, a conclusion should be attempted to be drawn by evaluating whether a history favoring the sexual abuse is present, together with positive and/or negative sings of the physical examination [9].

Most prominent complaint caused by anogenital warts is itching. Approximately one-third of these lesions will show spontaneous remission, even if not treated [8]. However, when it is considered that the itching is very severe or, as seen in our case, hand-anogenital region transmission process will persist; a treatment should be established to prevent the dissemination of the lesions and to eliminate the symptoms. The patients may be treated using chemical, destructive, or surgical methods, according to the localization and the size of the lesions. In this case, we preferred surgical excision, because the wart was histopathologically reported for forensic investigation; one of the lesions was above 1 cm; the patient did not show a response to the previous destructive therapy; and we predicted the formation of a greater number of scars using destructive methods. For the wart detected on the thumb, as a regression was obtained using cryotherapy before the admission to our hospital, we decided to monitorize it without administering an additional therapy. 

In this case, another important point is the development of LSK, which is a disease generally seen in adults, in a 9-year-old child secondarily to a wart. Although there were some reports about warts and LSK observed during the childhood, there was no case report that revealed concomitant presence of these two events in this age group [1–4]. While, in a case in whom anogenital warts were detected, it is easy to predict that this was developed secondarily to itching, the high efficacy of emotional status and psychological stress in the development of LSK may indicate a mood disorder resulting from a potential underlying sexual abuse. 

In this case, although warts that led to itching were surgically excised, episodes of itching related to probable LSK were decreased but not completely resolved. The use of topical and, in severe cases, systemic steroid therapy aiming the control of the itching and the resolution of the inflammation in the treatment of LSK should be avoided in the presence of infection with warts due to their ability to induce HPV viral genome transcription [10]. Therefore, in this case, steroid therapy was not given; the family was instructed to respect the hygienic rules and to keep anogenital area without humidity; the episodes of itching, which are exacerbated especially during the night, were prevented using oral antihistaminic therapy, and the monitorization showed a complete regression of LSK lesion.

Children in whom anogenital warts were detected, a thorough history and examination can help to rule out sexual abuse, and when etiology and transmission route are tried to be discovered, the child should be examined using a multidisciplinary approach for other possible concomitant cutaneous diseases.
